# Key amino acids in RNA polymerase and helicase proteins regulate RNA synthesis efficiency in porcine reproductive and respiratory syndrome virus

**DOI:** 10.1016/j.jbc.2025.110247

**Published:** 2025-05-16

**Authors:** Hui Li, Riteng Zhang, Honglin Xie, Yefei Zhou, Xinglong Wang

**Affiliations:** 1 College of Veterinary Medicine, Northwest A&F University, Yangling, Shaanxi, China; 2 School of Animal Science and Technology, Foshan University, Guangdong, China; 3 Department of Life Science, Nanjing Xiaozhuang University, Nanjing, Jiangsu, China

**Keywords:** PRRSV replicon, non-structural proteins (nsps), viral replication and transcription, adaptive mutations, RNA synthesis efficiency

## Abstract

Porcine reproductive and respiratory syndrome virus (PRRSV) exhibits rapid evolution due to its high mutation rate and frequent recombination, posing significant challenges for disease control. In this study, we investigated the molecular mechanisms underlying strain-specific variations in PRRSV replication phenotypes. Using reverse genetics and molecular biology approaches, we established a non-infectious replicon model that simulates PRRSV genomic replication and subgenomic (sg) mRNA transcription at the cellular level. This model enabled the evaluation of regulatory effects of viral non-structural proteins (nsps) and transcription-regulating sequences (TRSs) on viral replication and transcription, revealing the crucial roles of nsp9 and nsp12 in RNA synthesis. Furthermore, we developed a subgenomic replicon system (sg-Rep-PRRSV) driven by a minimal replication-transcription complex (mini-RTC) to investigate the impact of specific mutations in PRRSV replicase-associated proteins on viral RNA synthesis efficiency. Our findings demonstrated that mini-RTC components derived from XM-2020 exhibited significantly higher transcriptional driving efficiency compared to those from the GD strain (*p* < 0.01). Site-directed mutagenesis analysis identified critical amino acid residues contributing to differential RNA synthesis efficiency between strains: E141N, N416H, and S591A in nsp9, and S51D, L57T, and K349E in nsp10. These adaptive mutations likely modulate the catalytic conformations of RNA-dependent RNA polymerase (RdRp) and helicase, ultimately contributing to the distinct replication phenotypes observed among PRRSV strains. Our findings provide an insight into the molecular mechanisms underlying PRRSV evolution and adaptation, which have significant implications for mitigating future PRRS outbreak risks and maintaining the sustainable development of the swine industry.

Porcine reproductive and respiratory syndrome (PRRS), caused by porcine reproductive and respiratory syndrome virus (PRRSV), is a major economic threat to the global swine industry due to its rapid evolution and genetic diversity (1, 2, 3). PRRSV is an enveloped, positive-sense single-stranded RNA virus belonging to the Arteriviridae family, with a ∼15 kb genome that includes untranslated regions (UTRs) at both ends, which regulate viral RNA synthesis, subgenomic mRNA (sg mRNA) transcription, and protein translation (4, 5). The PRRSV genome consists of at least 10 open reading frames (ORFs), with ORF1a and ORF1b encoding replicase polyproteins that are processed into 16 non-structural proteins (nsps) (6, 7, 8). Among these, nsp9 (RNA-dependent RNA polymerase), nsp10 (helicase), and nsp11 (endoribonuclease) serve as key components of the viral replication–transcription complex (RTC), orchestrating genome replication and transcription (9, 10). Additionally, nsp12 and membrane scaffold proteins facilitate RTC assembly, ensuring efficient viral RNA synthesis (11, 12, 13, 14). The 3′ terminal quarter of the PRRSV genome encodes structural proteins, including GP5 and M, which form heterodimeric complexes essential for viral attachment and host cell invasion (9, 15). The nucleocapsid protein (N) binds genomic RNA (gRNA), protecting it from degradation and facilitating viral particle assembly (16).

Previous studies have demonstrated that the high mutation rate and frequent recombination events in PRRSV drive the rapid evolution of viral quasispecies (17, 18). Comparative analyses of predominant lineages have revealed significant differences among strains in replication efficiency and transcriptional regulation, suggesting that adaptive evolution of key viral proteins may constitute the molecular basis for these phenotypic variations (19, 20). Recent investigations focusing on specific mutations within nsps have confirmed that certain amino acid residues in replicase nsp9 and helicase nsp10 are closely associated with PRRSV virulence, pathogenicity, and replication efficiency (21, 22, 23). However, the underlying mechanisms by which these critical mutations regulate viral RNA synthesis and influence strain-specific replication dynamics remain unclear, largely due to the lack of suitable research models and technical approaches.

Non-infectious viral replicon systems serve as powerful tools for investigating RNA virus replication and transcription mechanisms. Compared to traditional reverse genetics approaches, replicon technology enables the simulation of viral RNA synthesis while reducing biosafety risks, facilitating dynamic analysis, and functional studies of key molecular events (24). Replicons allow targeted introduction of specific mutations and direct assessment of their effects on viral genome replication and transcription through reporter gene assays and RNA analysis, elucidating phenotypic effects and molecular mechanisms. Furthermore, replicons serve as effective platforms for screening and evaluating viral replicase inhibitors (25). Recent developments in replicon systems for positive-strand RNA viruses, including dengue virus, SARS-CoV-2, and MERS-CoV, have significantly enhanced our understanding of their replication mechanisms and accelerated antiviral drug development (26, 27, 28).

In this study, we established a non-infectious PRRSV replicon model based on reverse genetics, enabling simulation of viral genome replication and sg mRNA transcription at the cellular level. Furthermore, we developed a subgenomic replicon system and systematically evaluated the impact of key nsp mutations on RNA synthesis by analyzing amino acid differences between representative strains from two major PRRSV-2 lineages, L1.8 and L8.7. These findings establish a scientific foundation for guiding the design of vaccines and the optimization of comprehensive control strategies.

## Results

### Construction and validation of a PRRSV replicon system

**Figure 1:**
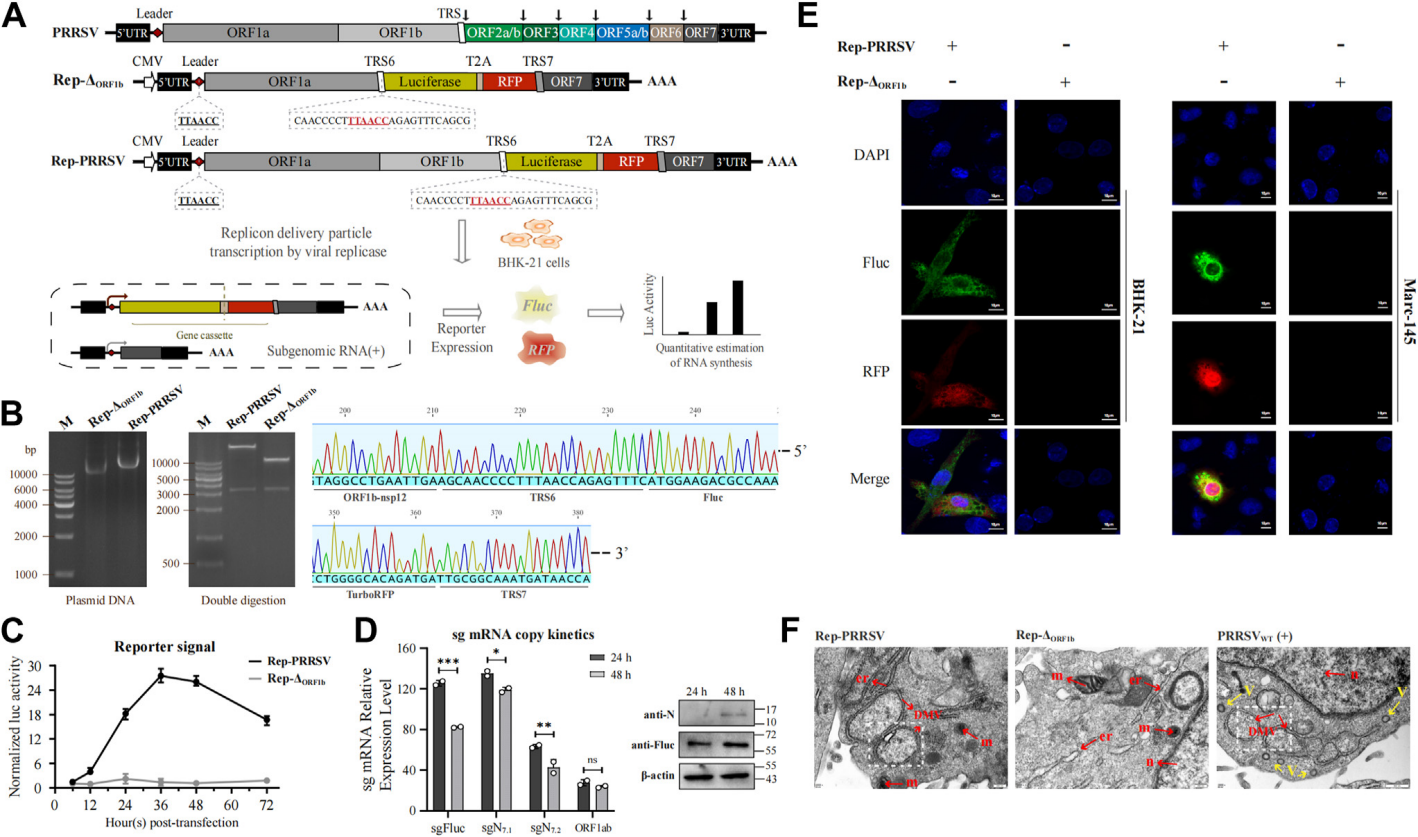
**Construction and transcriptional analysis of the PRRSV replicon.***A*, schematic diagram of reporter replicon genome structure, replication, and sg mRNA production. ORF2-6 in the wild-type viral genome were deleted and replaced with the Fluc-T2A-RFP gene cassette. *B*, gel electrophoresis, restriction enzyme digestion (NotI and AscI), and sequencing analysis of Rep-PRRSV and Rep-ΔORF1b replicon plasmids. *C*, replication kinetics of Rep-PRRSV. BHK-21 cells were co-transfected with pRL-TK (250 ng) and Rep-PRRSV (2.5 μg), harvested at different time points, and firefly luciferase activity was measured and normalized to Renilla luciferase reading. *D*, RT-qPCR analysis of sg mRNA levels for Fluc, N, and ORF1ab in BHK-21 cells transfected with Rep-PRRSV, normalized to β-actin gene expression. *E*, Rep-PRRSV-transfected BHK-21 cells were analyzed at 36 h post-transfection (hpt) by confocal microscopy. Fluc was detected using an anti-Fluc mAb and goat-anti-mouse IgG conjugated with Alexa Fluor 488. Nuclei were stained with Hoechst. *F*, electron microscopy analysis of double-membrane vesicle structures induced by Rep-PRRSV transfection at 24 hpt. Two-way ANOVA was used to determine statistical significance. ∗∗∗*p* < 0.001; ∗∗*p* < 0.01; ∗*p* < 0.05; ns, not significant.

A non-infectious PRRSV replicon (Rep-PRRSV) was constructed *via* reverse genetics, retaining pp1a, pp1b, and N protein while deleting ORF2-6, enabling simulation of viral RNA synthesis in host cells and maintaining RNA replication activity while preventing progeny virus production. A firefly luciferase (Fluc)-T2A-red fluorescent protein (RFP) reporter was inserted downstream of transcription-regulating sequence 6 (TRS6), with poly(A) tail, hepatitis delta virus ribozyme (HdvRz), and bovine growth hormone (BGH) terminator at the 3′ terminus. Furthermore, the post-translationally processed products of ORF1b translation-nsp9, nsp10, nsp11, and nsp12-constitute core components of the PRRSV replicase and play critical roles in viral genomic replication and transcription, while also serving as key hub proteins connecting the PRRSV nsp interaction network. Therefore, we constructed a replication-deficient control replicon (Rep-ΔORF1b) with ORF1b deletion based on Rep-PRRSV (Fig. 1*A*). The constructed DNA fragments and plasmids were verified by agarose gel electrophoresis (Fig. 1*B*).

Rep-PRRSV demonstrated significant Fluc expression at 12 h post-transfection (hpt) compared to Rep-ΔORF1b, with activity declining after 36 hpt due to cytotoxicity (Fig. 1*C*). RT-qPCR analysis demonstrated peak transcription levels of subgenomic (sg) mRNAFluc, sg mRNA7.1, and sg mRNA7.2 at 24 hpt with slight reduction at 48 hpt, while viral N protein and Fluc expression progressively accumulated as evidenced by Western blot (Fig. 1*D*). Indirect immunofluorescence revealed distinct co-localization of Fluc and RFP, validating the dual reporter system for monitoring viral RNA synthesis dynamics (Fig. 1*E*). Transmission electron microscopy showed double-membrane vesicles in Rep-PRRSV-transfected MARC-145 cells at 24 hpt, consistent with replication and transcription complex (RTC) assembly sites in infectious PRRSV, whereas Rep-ΔORF1b exhibited no apparent membrane reorganization (Fig. 1*F*).

This replicon system effectively mimics viral genome replication and transcription, providing a tool for studying PRRSV RNA synthesis mechanisms and nsp mutations.

### Differential regulation of reporter gene expression by sistinct TRS elements

**Figure 2:**
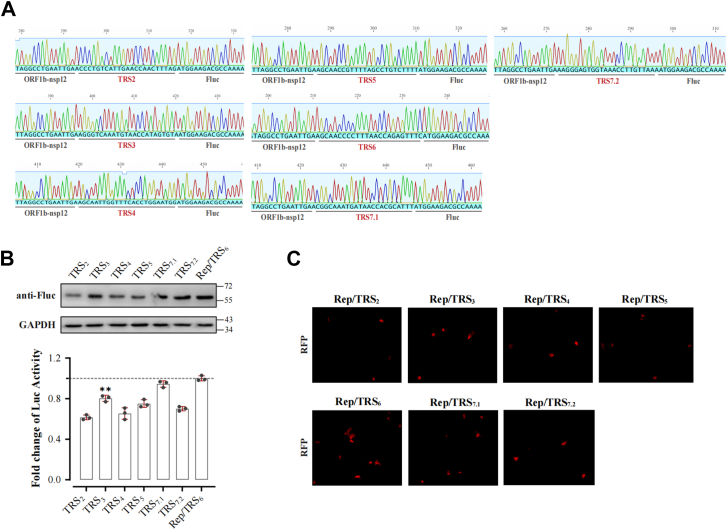
**Evaluation of transcriptional regulatory efficiency of TRSs in Rep-PRRSV.***A*, replicon plasmids containing transcriptional units with different transcription-regulating sequences B (TRSB) (TRS2: ACCCTGTCATTGAACCAACTTTAG, TRS3: AGGGTCAAATGTAACCATAGTGTA, TRS4: AGCAATTGGTTTCACCTGGAATGG, TRS5: AGCAACCGTTTTAGCCTGTCTTTT, TRS6: AGCAACCCTTTAACCAGAGTTTC, TRS7.1: ACGGCAAATGATAACCACGCATTT, TRS7.2: AAGGGAGTGGTAAACCTTGTTAAA) inserted upstream of the Fluc-T2A-RFP reporter were constructed using the Rep-PRRSV backbone. *B*, Luciferase activity and (*C*) RFP fluorescence intensity was measured at 36 h post-transfection to assess TRS regulatory efficiency. One-way ANOVA was used to determine statistical significance. ∗∗∗*p* < 0.001; ∗∗*p* < 0.01; ∗*p* < 0.05; ns, not significant.

To assess the contribution of transcription-regulating sequences (TRS) as cis-acting elements in PRRSV transcriptional regulation, replicon plasmids containing specific TRS elements were constructed based on Rep-PRRSV, these sequences exhibit considerable heterogeneity (Fig. 2*A*). In MARC-145 cells at 36 hpt, all TRS exhibited different levels of transcriptional activity. Using TRS6 (M gene) as the reference (value = 1), we found that TRS7.1 (N gene) showed comparable transcriptional activity with no significant difference from TRS6. In contrast, while TRS3 displayed higher expression levels than TRS2, TRS4, and TRS5, its activity remained significantly lower than TRS6 (*p* < 0.05) (Fig. 2*B*). Quantitative analysis of RFP fluorescence intensity confirmed the differential transcriptional activities of these TRS elements (Fig. 2*C*).

Sequence analysis revealed complete complementarity between TRS6's 13-nt conserved sequence (5′-acccctttaacca-3′) and the leader sequence at the viral genome's 5′ terminus. This precise pairing between the leader TRS (TRSL) and the complementary TRS6 sequence (cTRS6) likely facilitates template switching, enhancing sg mRNA synthesis and release efficiency at this site. These results suggest PRRSV modulates structural gene transcription through specific TRS nucleotide compositions, with transcriptional efficiency correlating with the degree of complementarity between the TRS and the leader sequence, as evidenced by the strong performance of TRS6 which exhibits perfect complementarity with the leader sequence.

### To regulatory effects of PRRSV non-structural proteins on replicon transcription

**Figure 3:**
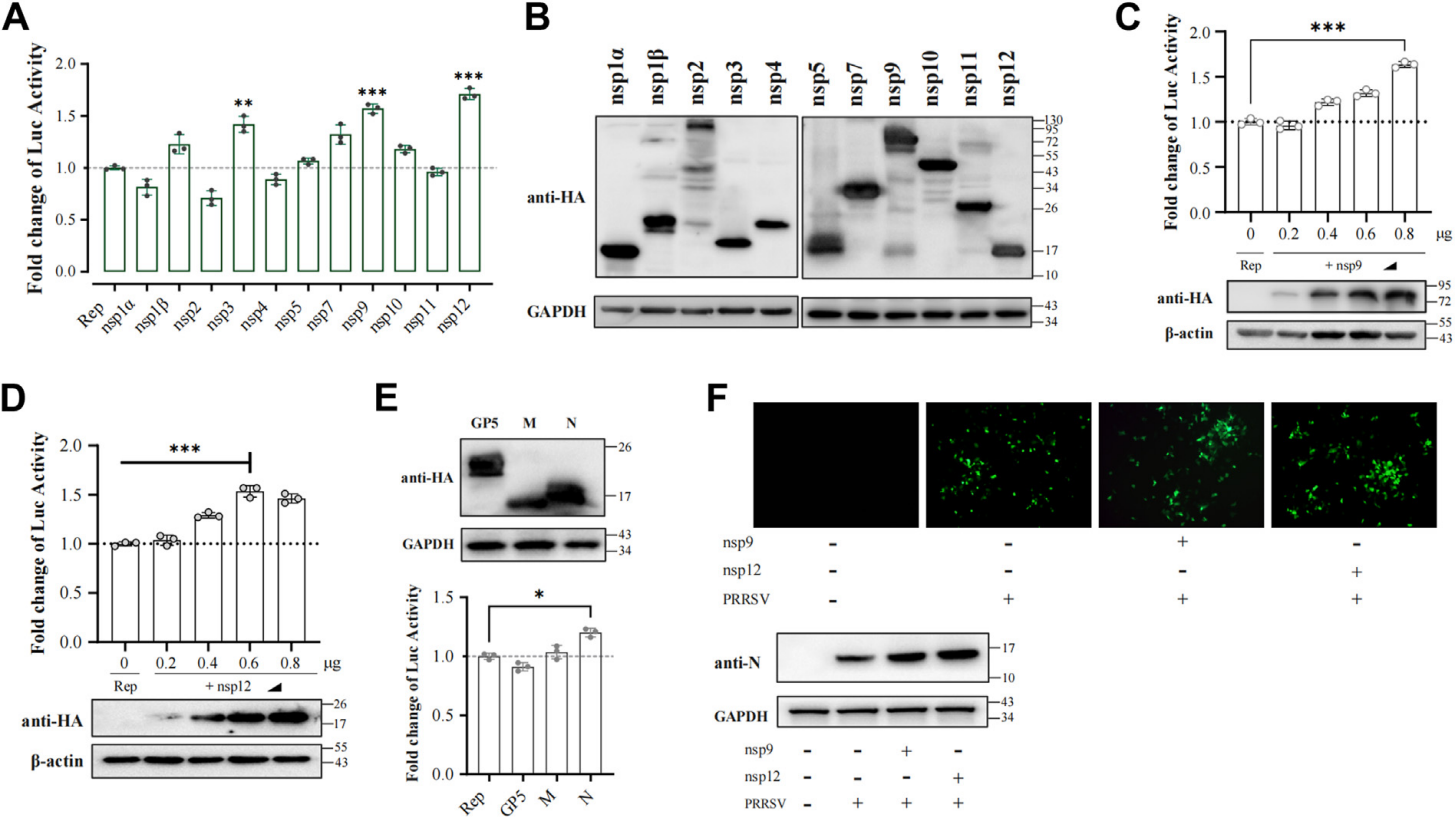
**Regulatory effects of PRRSV non-structural and structural proteins on replicon transcription.***A*, BHK-21 cells were co-transfected with Rep-PRRSV (1 μg) and plasmids expressing individual PRRSV nsps (0.5 μg), and Fluc activity was measured at 36 hpt to assess their effects on replicon transcription. *B*, PRRSV nsps protein expression was analyzed by Western blot 36 hpt. *C* and *D*, increasing concentrations of nsp9-HA and nsp12-HA plasmids were co-transfected with Rep-PRRSV into BHK-21 cells, and luciferase activity was measured at 36 hpt. *E*, effect of GP5, M, and N overexpression on Rep-PRRSV transcriptional activity. *F*, Analysis of nsp9 and nsp12 overexpression effects on rHP-PRRSV/SD16/TRS6-EGFP replication. MARC-145 cells were transfected with nsp9-HA or nsp12-HA plasmids for 24 h, followed by infection with rHP-PRRSV/SD16/TRS6-EGFP (MOI 0.1). Cells were harvested 24 h later, and viral replication was analyzed by Western blot. Data represent the mean ± SD of three independent experiments. ∗∗∗*p* < 0.001; ∗∗*p* < 0.01; ∗*p* < 0.05; ns, not significant compared to the indicated control samples.

To investigate the role of PRRSV non-structural proteins (nsps) in viral RNA synthesis, nsp expression plasmids were co-transfected with Rep-PRRSV into BHK-21 cells. While most nsps showed minimal impact, nsp9 and nsp12 overexpression significantly enhanced reporter gene transcription (*p* < 0.001) (Fig. 3, *A* and *B*). Western blot analysis confirmed dose-dependent activation of replicon transcription with increasing nsp9 and nsp12 expression levels (Fig. 3, *C* and *D*).

Assessment of structural proteins revealed that only N protein exhibited modest promotional effects (*p* < 0.05), while GP5 and M protein expression demonstrated no detectable effect on Rep-PRRSV transcription efficiency (Fig. 3*E*), indicating that viral RNA synthesis is predominantly regulated by nsps rather than structural proteins.

Validation using the recombinant virus rHP-PRRSV/SD16/TRS6-EGFP confirmed that nsp9 and nsp12 overexpression enhanced viral RNA synthesis during infection, corroborating the replicon-based observations (Fig. 3*F*), which supports their critical regulatory roles in viral genome replication and transcription.

### To construction and activity analysis of PRRSV mini-replicon system

**Figure 4:**
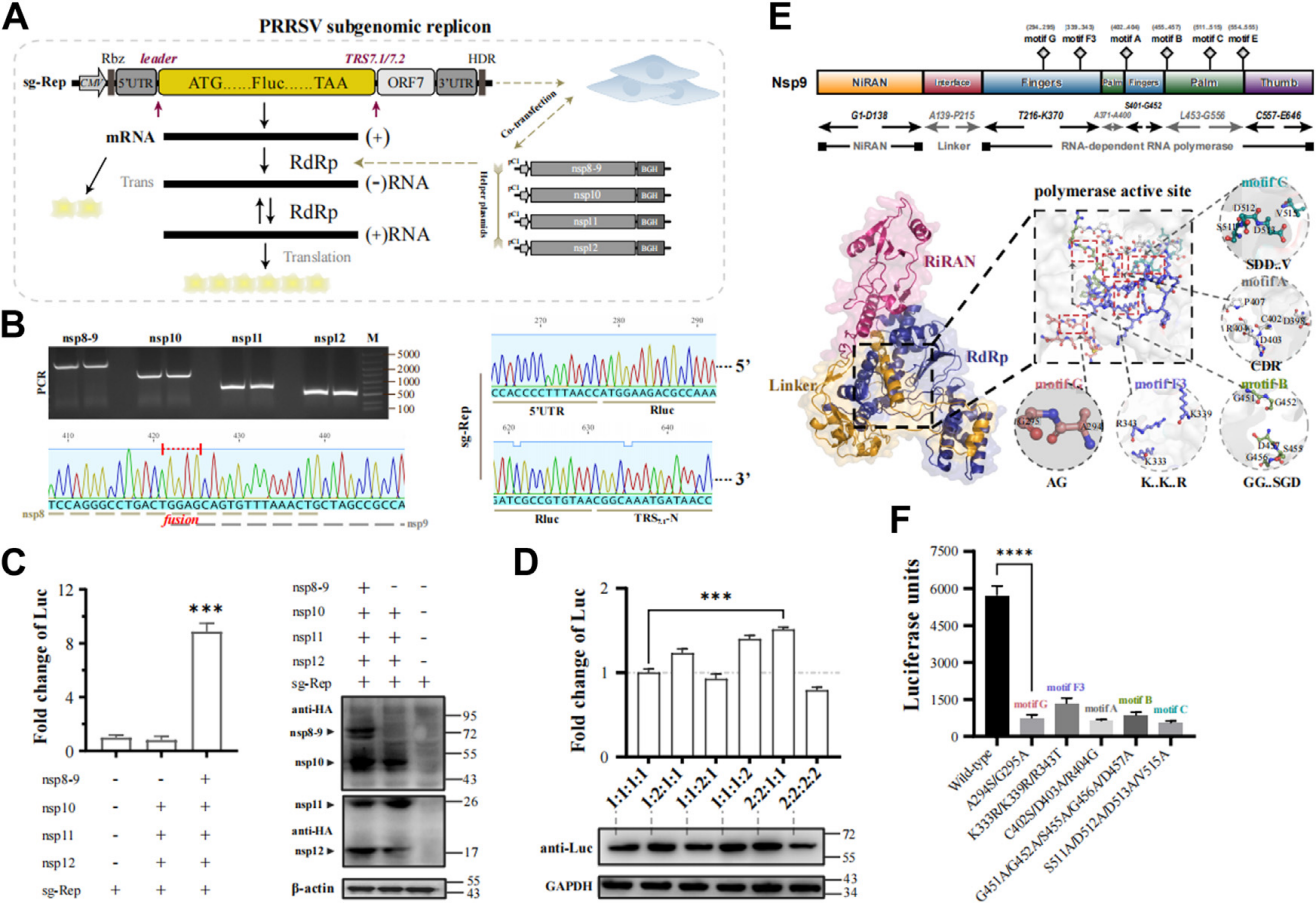
**Construction and characterization of subgenomic replicon sg-Rep-PRRSV.***A*, schematic diagram of the sg-Rep-PRRSV subgenomic replicon containing 5′ and 3′UTRs, leader TRS, Fluc reporter, and ORF7. *B*, construction and sequencing validation of sg-Rep-PRRSV and mini-RTC system plasmids. *C*, BHK-21 cells were co-transfected with the indicated plasmids, and Fluc activity and protein expression were analyzed by luciferase assay and Western blot at 36 h post-transfection (hpt). *D*, analysis of sg-Rep-PRRSV-Fluc expression under different ratios of nsp8-9, nsp10, nsp11, and nsp12. *E*, AlphaFold two was used to model the 3D structure of PRRSV nsp9, showing specific functional regions (NiRAN, fingers, palm, and thumb of RdRp) and the spatial positions of conserved motifs, as visualized using PyMOL. *F*, activity assessment of nsp9 conserved functional domain mutants using the sg-Rep-PRRSV system. Bars represent mean ± SD. ∗∗∗∗ *p* < 0.0001; ∗∗∗*p* < 0.001; ∗∗*p* < 0.01; ∗*p* < 0.05; ns, not significant.

Despite successful construction of a full-length PRRSV replicon containing all nsp genes, its extensive sequence limits functional studies of replicase mutations. We developed a minimal PRRSV replicon based on SARS-CoV-2 mini-replicon principles (29, 30). A mini-RTC system was engineered comprising nsp9 (RdRp), nsp10, nsp11, and nsp12, wherein the ribosomal frameshift site between nsp8-9 was eliminated to generate a fusion protein (31). The sg-Rep-PRRSV retained only 5'/3′UTRs, leader TRS, and ORF7 sequences, with Fluc reporter gene inserted upstream of ORF7 (Fig. 4*A*). The constructed DNA fragments and plasmids were verified by agarose gel electrophoresis (Fig. 4*B*).

Co-transfection of mini-RTC components with sg-Rep-PRRSV in BHK-21 cells enhanced Fluc transcription by 8.9-fold (*p* < 0.001) compared to sg-Rep-PRRSV alone. Western blot analysis confirmed efficient expression of all nsps (Fig. 4*C*). Optimal activity was achieved at a molar ratio of nsp 8 to 9: nsp 10: nsp 11: nsp 12 of 2:2:1:1 (Fig. 4*D*), suggesting specific stoichiometric requirements for complex assembly. Further site-directed mutations were conducted on the conserved functional domains of nsp9 (such as SDD.V→AAA.A) and incorporated into the mini-RTC system. For the F3 motif (K.K.K), we implemented a strategic approach utilizing K→R and K→T substitutions rather than complete alanine replacements. This sophisticated design enabled us to specifically investigate the effects of both charge properties (preserved in K→R but altered in K→T) and structural elements on RdRp function, as the lysine residues within the F3 motif critically interact with the RNA backbone for precise template positioning. Our results demonstrated that all mutants impaired the transcriptional activity of the replicon to varying extents, further emphasizing the essential nature of these key motif residues in maintaining RdRp functionality and orchestrating RNA synthesis processes (Fig. 4, *E* and *F*).

### XM-2020 mini-RTC significantly enhances sg-Rep-PRRSV (GD) transcription efficiency

**Figure 5:**
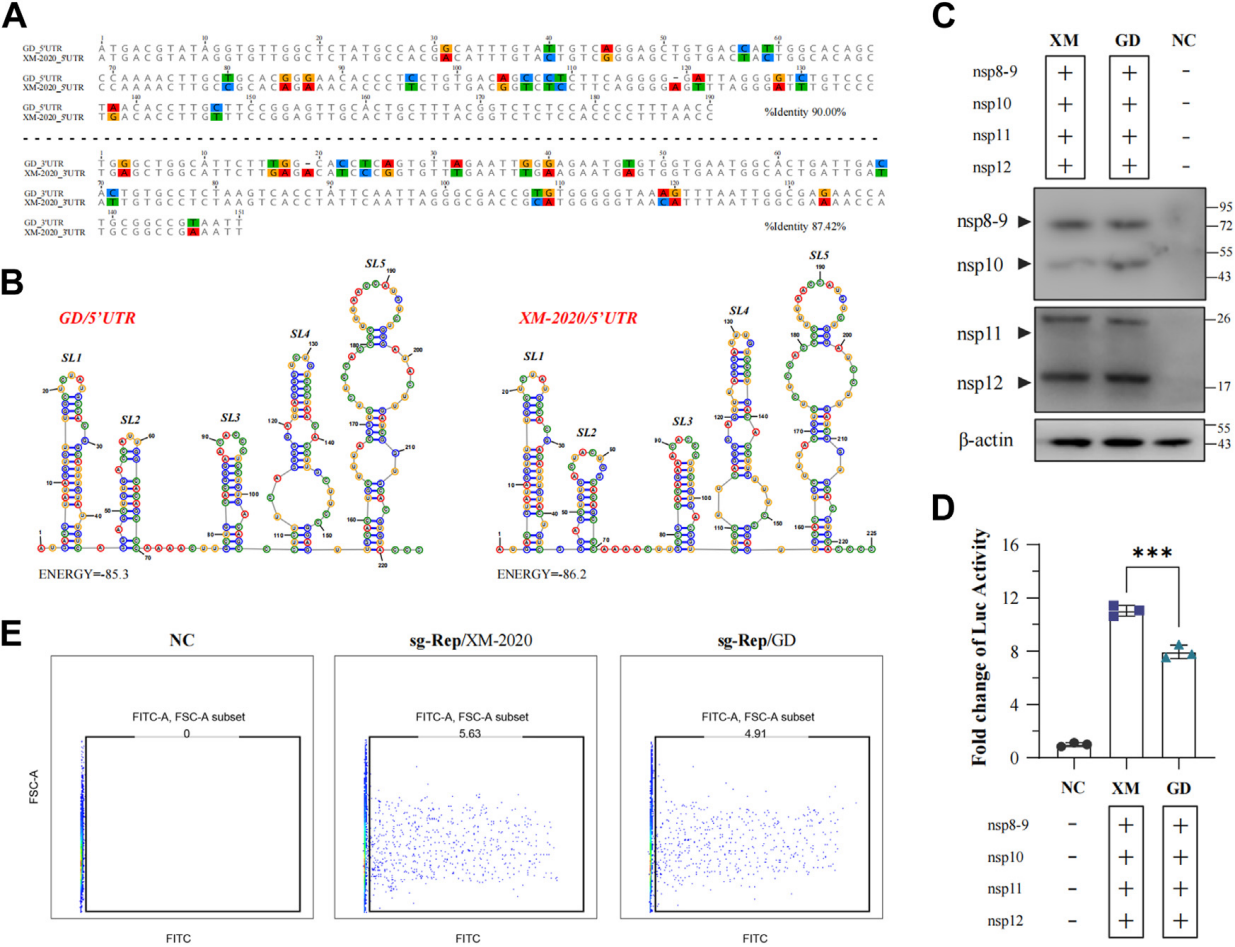
**Evaluation of the effect of GD- and XM-2020-derived mini-RTC components on sg-Rep-PRRSV(GD) transcription.***A*, sequence alignment and (*B*) predicted secondary structures of the 5′ and 3′UTRs from PRRSV GD and XM-2020. *C*, expression of nsps, (*D*) Fluc activity assay, and (*E*) percentage of Fluc-positive cells in BHK-21 cells co-transfected with sg-Rep-PRRSV and mini-RTC components derived from either GD or XM-2020 at 36 hpt. Data represent the mean ± SD of three independent experiments. ∗∗∗*p* < 0.001; ∗∗*p* < 0.01; ∗*p* < 0.05; ns, not significant.

PRRSV genomic RNA synthesis primarily relies on the conserved 5′ and 3′ untranslated regions (UTRs). Considering the frequent genomic recombination phenomena in PRRSV, it was hypothesized that RTC complexes from different strains possess the ability to “cross-recognize” and utilize heterologous UTRs, thereby driving the synthesis of chimeric sg mRNAs. This study selected GD and XM-2020 strains for comparative analysis. The nucleotide homology between the two viruses' 5′ and 3′ UTRs was 90.00% and 87.42%, respectively, and the predicted RNA secondary structures were also highly similar (Fig. 5, *A* and *B*). To verify this hypothesis, we constructed a subgenomic replicon sg-Rep-PRRSV (GD) based on GD strain UTRs and introduced mini-RTC components from both GD and XM-2020 to examine the effect of heterologous RTC on replicon transcription activity. Western blot results showed that nsps from both XM-2020 and GD were effectively expressed in transfected BHK-21 cells, with comparable expression levels (Fig. 5*C*). Luciferase activity detection demonstrated that XM-2020 mini-RTC not only effectively drove the transcription of sg-Rep-PRRSV (GD) but also exhibited significantly higher Fluc activity values and proportions of Fluc-positive cells compared to GD's own mini-RTC (Fig. 5, *D* and *E*).

The above research supports that PRRSV trans-lineage mini-RTC components possess the ability to “cross-recognize” and utilize “non-self” viral RNA initiation sequences, a finding consistent with Gao *et al.*'s study showing that PRRSV-2 maintained its replication after 5′UTR replacement (32). Although XM-2020 and GD belong to different lineages, the former's replicase core components can effectively cross-recognize GD strain's cis-acting elements and initiate subgenomic replicon transcription, with efficiency significantly superior to GD itself. This suggests that XM-2020 replicase-related proteins may have accumulated certain adaptive mutations during evolution, enabling them to achieve more efficient and broader RNA synthesis activity and substrate adaptability, which may be the molecular basis for its stronger replication advantage compared to the GD strain.

### Impact of characteristic nsp9 mutations on replication-transcription efficiency

**Figure 6:**
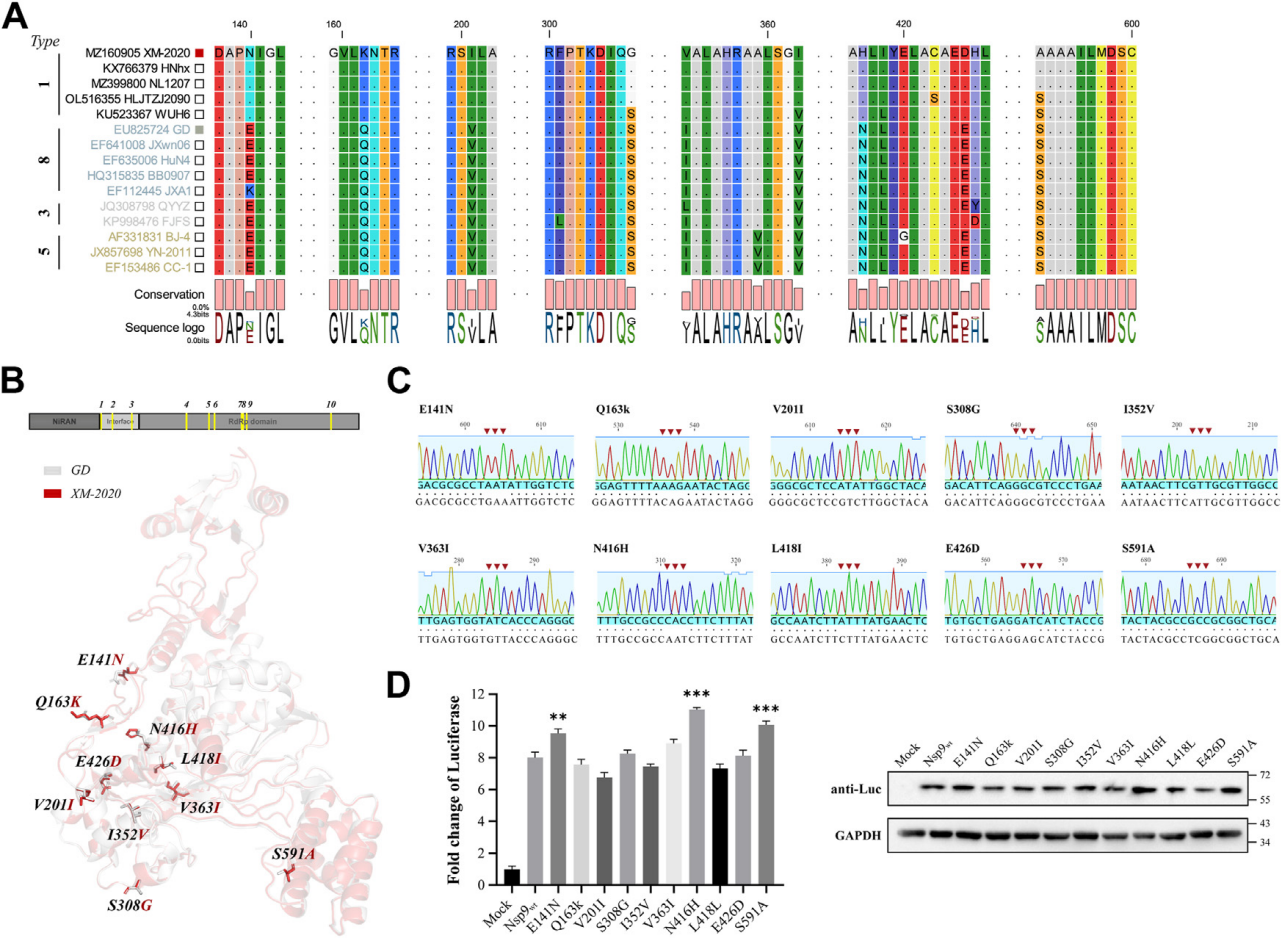
**Effect of nsp9 characteristic mutations on sg-Rep-PRRSV transcription efficiency.***A*, sequence alignment of nsp9 from PRRSV GD and XM-2020 strains, highlighting amino acid differences. *B*, AlphaFold 2-predicted 3D structure of nsp9 with selected mutations highlighted in stick representation in PyMOL. *C*, sequencing results of nsp8-9 expression plasmids carrying single point mutations. *D*, BHK-21 cells were co-transfected with sg-Rep-PRRSV, nsp9 mutants, and other mini-RTC components (nsp10–12), and Fluc activity was measured at 36 hpt. Data represent the mean ± SD of three independent experiments. ∗∗∗*p* < 0.001; ∗∗*p* < 0.01; ∗*p* < 0.05; ns, not significant.

To elucidate the underlying mechanism of differential RNA synthesis efficiency between XM-2020 and GD mini-RTC systems, comparative sequence analysis of nsp9 from both strains was performed. This analysis revealed multiple amino acid variations, including E141N, Q163K, V201I, S308G, I352V, V363I, N416H, L418I, E426D, and S591A (Fig. 6*A*). To assess the functional impact of these substitutions on PRRSV RNA synthesis, a series of nsp8-9 expression plasmids harboring specific amino acid mutations were generated based on the GD strain mini-RTC backbone (Fig. 6*C*). These constructs were subsequently co-transfected with other mini-RTC components and sg-Rep-PRRSV into BHK-21 cells. At 36 h post-transfection (hpt), transcriptional efficiency was quantified *via* Fluc activity assay. As illustrated in Figure 6*D*, the introduction of E141N, N416H, or S591A substitutions significantly enhanced replicon transcription levels compared to GD nsp9WT. Conversely, mutations such as Q163K and V201I exhibited no significant alteration in Fluc expression.

AlphaFold 2-based structural modeling of nsp9 demonstrated that the N416H mutation resides within the “fingers” domain of RdRp, suggesting that the resultant polarity shift may modulate the dynamic conformation of critical catalytic sites. Meanwhile, the S591A substitution, located on the “thumb” domain surface, represents a transition from a hydrophilic to a hydrophobic residue, potentially influencing RdRp-template RNA interactions through alterations in the local hydrophobic microenvironment (Fig. 6*B*). While these findings provide preliminary insights into the molecular mechanisms by which key nsp9 amino acid substitutions regulate RNA synthesis efficiency, the precise mode of action requires further validation through comprehensive biochemical and structural biology investigations.

### Impact of characteristic nsp10 mutations on replication-transcription efficiency

**Figure 7:**
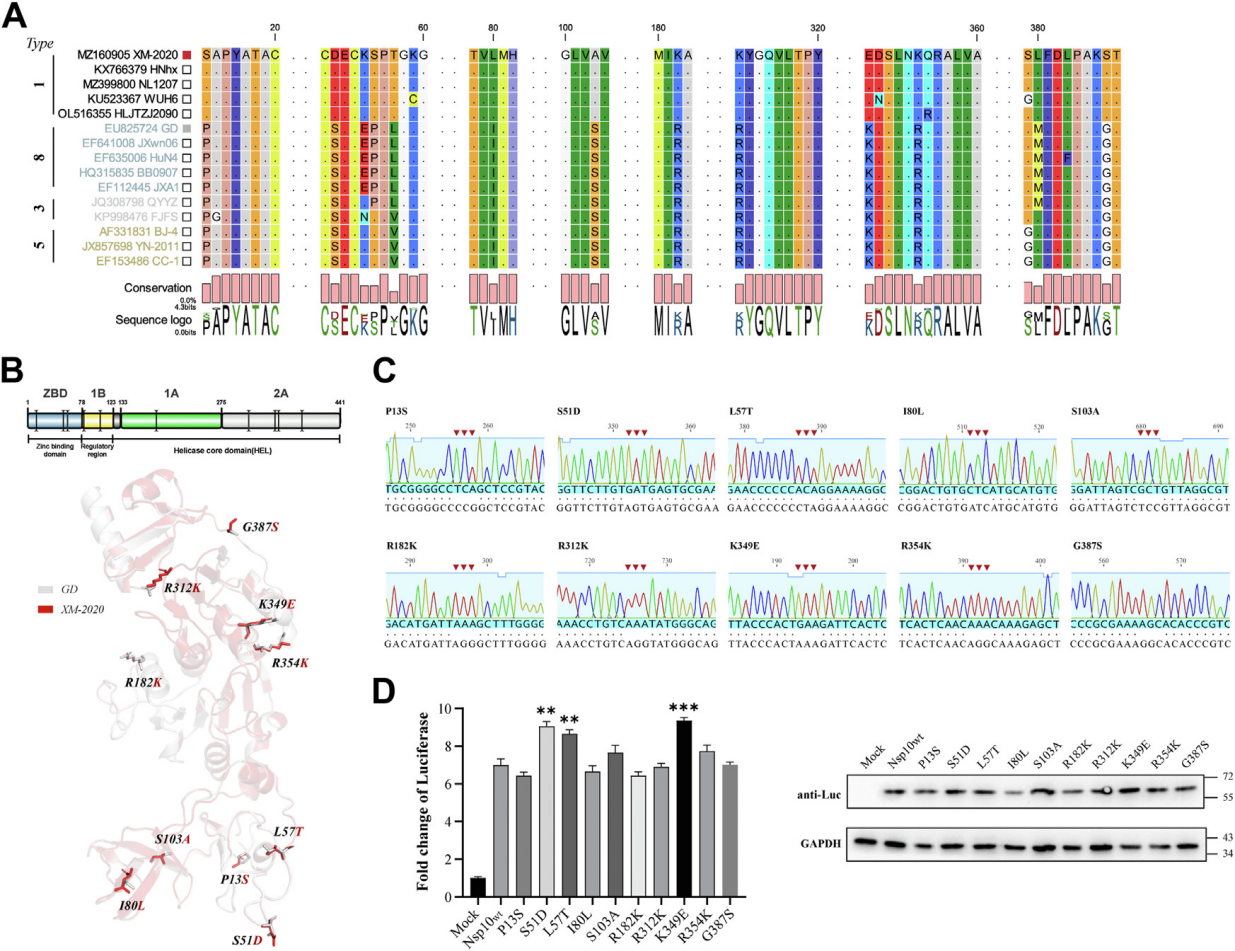
**Effect of nsp10 characteristic mutations on sg-Rep-PRRSV transcription efficiency.***A*, sequence alignment of nsp10 from PRRSV GD and XM-2020 strains, highlighting amino acid differences. *B*, AlphaFold 2-predicted 3D structure of nsp10 with selected mutations highlighted in stick representation in PyMOL. *C*, sequencing results of nsp10 expression plasmids carrying single point mutations. *D*, BHK-21 cells were co-transfected with sg-Rep-PRRSV, nsp10 mutants, and other mini-RTC components, and Fluc activity was measured at 36 hpt. Data are presented as mean ± SD, representing three independent experiments. ∗∗∗*p* < 0.001; ∗∗*p* < 0.01; ∗*p* < 0.05; ns, not significant.

To elucidate the influence of specific nsp10 amino acid residues on PRRSV mini-replicon transcriptional activity, a comprehensive sequence comparison between GD and XM-2020 strains was performed. This analysis identified multiple variant positions including P13S, S51D, L57T, I80L, S103A, R182K, R312K, K349E, R354K, and G387S (Fig. 7*A*). Subsequently, a series of nsp10 expression plasmids harboring individual point mutations were generated (Fig. 7*C*) and co-transfected with additional mini-RTC components and sg-Rep-PRRSV into BHK-21 cells. Quantitative Fluc activity assessment revealed that S51D, L57T, and K349E substitutions significantly enhanced replicon transcription efficiency, indicating their potential regulatory roles in nsp10 catalytic function (Fig. 7*D*). Structural analysis using AlphaFold two demonstrated that S51D and L57T mutations reside within the zinc-binding domain (ZBD) (Fig. 7*B*). Notably, the L57T substitution represents a transition from a hydrophobic to a polar residue, potentially increasing ZBD flexibility and thereby facilitating dynamic RNA substrate interactions. The K349E mutation, positioned within the helicase core domain (HEL 2A), introduces a charge reversal from positive to negative, which may alter the local electrostatic environment proximal to the ATP-binding pocket, consequently modulating ATP hydrolysis efficiency.

### Impact of characteristic nsp12 mutations on replication-transcription efficiency

**Figure 8:**
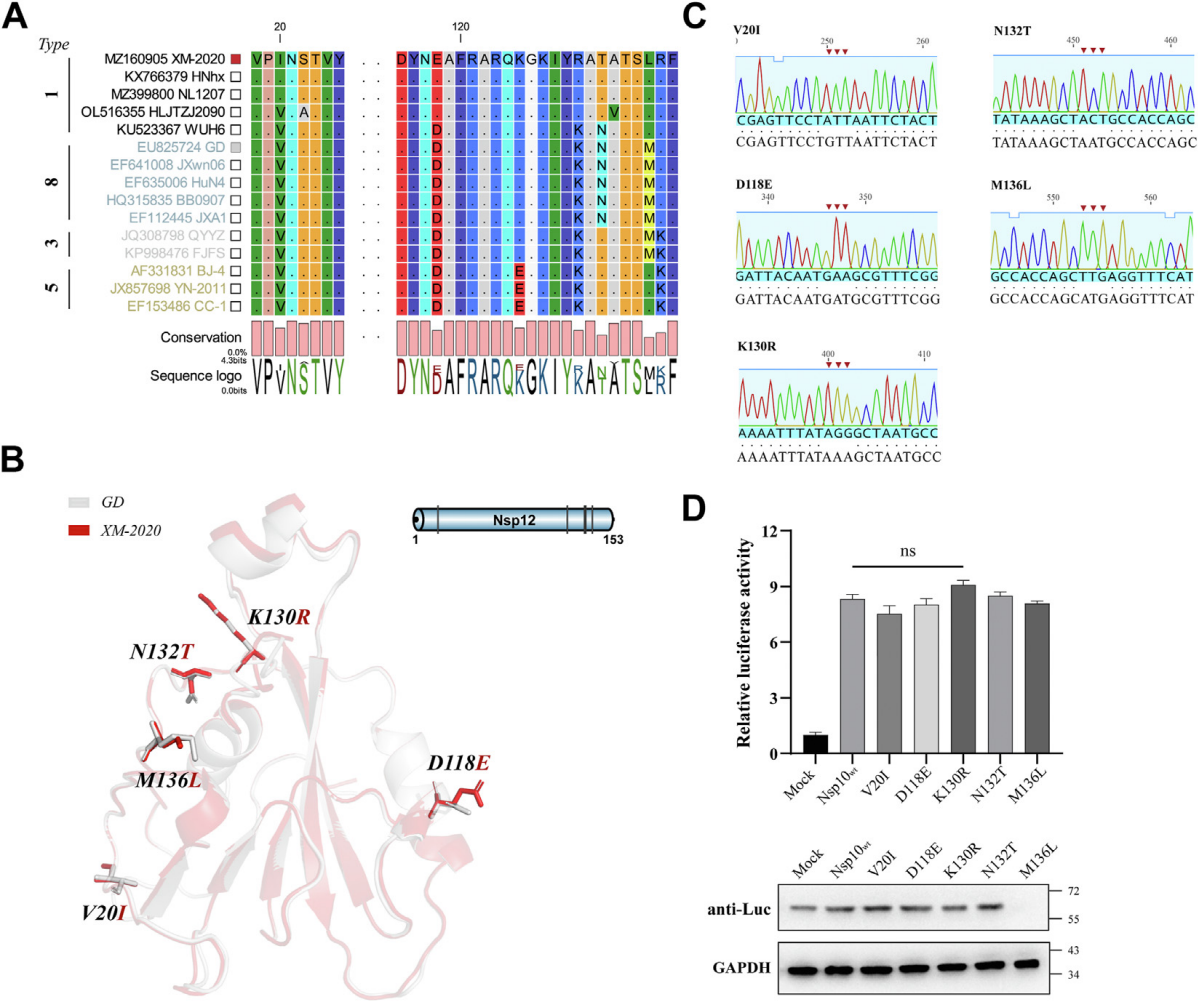
**Effect of nsp12 characteristic mutations on sg-Rep-PRRSV transcription efficiency.***A*, sequence alignment of nsp12 from PRRSV GD and XM-2020 strains, highlighting amino acid differences. *B*, AlphaFold 2-predicted 3D structure of nsp12 with selected mutations highlighted in stick representation in PyMOL. *C*, sequencing results of nsp12 expression plasmids carrying single point mutations. *D*, BHK-21 cells were co-transfected with sg-Rep-PRRSV, nsp12 mutants, and other mini-RTC components, and Fluc activity was measured at 36 hpt. Data are mean ± SD from at least three biological replicates. ∗∗∗*p* < 0.001; ∗∗*p* < 0.01; ∗*p* < 0.05; ns, not significant.

To assess the contribution of specific nsp12 amino acid residues to viral replication and transcription efficiency, a comparative sequence analysis between GD and XM-2020 strains was conducted. This analysis identified several variant positions, including V20I, D118E, K130R, N132T, and M136L (Fig. 8, *A*–*C*). Individual nsp12 mutants were co-transfected with additional mini-RTC components and sg-Rep-PRRSV into BHK-21 cells, followed by quantitative assessment of Fluc activity. As illustrated in Figure 8*D*, none of these substitutions induced significant alterations in Fluc expression levels, suggesting that these variant residues do not substantially influence replicon transcriptional efficiency.

## Discussion

Due to extensive genetic variation and frequent recombination, PRRSV strains exhibit diverse phenotypes in host cell tropism, replication dynamics, and pathogenicity (33, 34, 35). Previous studies have demonstrated that specific amino acid mutations in nsp9 and nsp10 significantly influence PRRSV replication efficiency and virulence (22, 36). However, the molecular mechanisms by which these key mutations modulate strain-specific phenotypes through regulation of viral RNA synthesis remain to be elucidated. Comparative studies of representative strains XM-2020 and GD from two major PRRSV-2 lineages (L1.8 and L8.7) in porcine alveolar macrophages (PAMs) revealed significant differences in their replication kinetics (19). To investigate the molecular basis of these differences, we established PRRSV replicon and mini-replicon systems with autonomous RNA synthesis activity. These systems effectively recapitulate viral genome replication and transcription processes, providing robust tools for systematically investigating the molecular determinants of differential replication-transcription efficiency between PRRSV strains and exploring crucial elements in viral RNA synthesis. Our analysis revealed that mutations E141N, N416H, and S591A in nsp9, as well as S51D, L57T, and K349E in nsp10, enhanced replicon sg mRNA transcription efficiency to varying degrees, while nsp12 mutations exhibited no significant effect on replicon activity. Furthermore, we demonstrated that PRRSV RNA synthesis is predominantly regulated by coordinated nsp functions, with structural proteins playing minimal roles in this process. These findings advance our understanding of PRRSV replication regulatory mechanisms.

For RNA viruses, spatiotemporal regulation of RNA synthesis represents a critical process during the infection cycle (37). While moderate enhancement of RNA-dependent RNA polymerase (RdRp) catalytic rate facilitates rapid viral infection establishment and progeny production, thereby improving population transmission fitness, excessive mutation rates may introduce deleterious mutations into the viral genome, potentially compromising population stability (38, 39, 40, 41, 42, 43). Therefore, balancing RNA synthesis rate with replication fidelity presents a crucial evolutionary challenge for viral RdRps. Previous studies have demonstrated that the dynamic transition between “open” and “closed” conformations of the RdRp catalytic center is essential for regulating polymerase activity, primarily through subtle conformational changes in conserved residues (particularly Asp) within the palm domain's motif A (44). In SARS-CoV-2, the RdRp (nsp12) contains a unique Ala547 in motif F, replacing the conserved Glu residue found in most viral RdRps, while its motif C sequence is Ser-Asp-Asp (SDD) rather than Gly-Asp-Asp (GDD). Site-directed mutagenesis studies revealed that A547E and S759G mutations altered nsp12 polymerization rates by 2-fold and 7-fold, respectively, with A547E increasing fidelity while S759G decreased it (41).

Notably, coronaviruses achieve proofreading functionality through nsp14's 3′-5′ exonuclease activity, maintaining an overall base substitution frequency of 10^−6^-10^−7^, while ensuring high replication efficiency, thus establishing a balance between rapid replication and fidelity (45, 46). In contrast, arteriviruses like PRRSV, lacking identified proofreading enzymes, may rely more heavily on fine-tuning RdRp dynamic activity for adaptive evolution. Our research demonstrates that distinct TRS elements within the PRRSV genome contribute to viral transcription regulation, with precise pairing between the leader TRS (TRSL) and the complementary TRS6 sequence (cTRS6), potentially enhancing sg mRNA synthesis efficiency. Furthermore, site-directed mutagenesis of conserved functional domains in nsp9 using mini-RTC abolished replicon transcription activity, demonstrating the essential role of key motif residues in maintaining RdRp function and mediating viral RNA synthesis. These findings indicate that PRRSV achieves precise regulation of viral structural gene transcription through modulation of TRS element sequence features and conserved motif residues.

PRRSV genomic RNA synthesis is predominantly dependent on conserved 5′ and 3′ untranslated regions (UTRs). Given the frequent genomic recombination observed in PRRSV, we hypothesized that RTC complexes from different strains possess the ability to recognize and utilize heterologous UTRs across strain boundaries, thereby facilitating the synthesis of chimeric sg mRNAs. Our findings validate this hypothesis, demonstrating that PRRSV cross-lineage mini-RTC components exhibit trans-recognition capability for non-cognate viral RNA initiation sequences. This observation is consistent with previous studies showing that PRRSV-2 maintains replication capability following 5′UTR substitution (32).

Although XM-2020 and GD belong to distinct lineages, the replicase core components of XM-2020 efficiently recognize GD strain cis-acting elements and initiate subgenomic replicon transcription with significantly higher efficiency than the homologous GD components. These results suggest that XM-2020 replicase-associated proteins may have acquired specific adaptive mutations during evolution, conferring enhanced RNA synthesis activity and broader substrate recognition. These molecular characteristics likely contribute to its replication advantage over the GD strain.

Previous studies have demonstrated that replacing non-structural protein regions of nsp9 and nsp10 from high-virulence PRRSV strains with corresponding regions from low-virulence strains can significantly enhance the replication efficiency and pathogenicity of the latter (21). Reverse genetics evidence supports the critical role of specific amino acid mutations in nsp9 in modulating viral characteristics. Notably, mutations at positions S519T and T544A significantly improved high-pathogenicity PRRSV (HP-PRRSV) replication efficiency in porcine alveolar macrophages (PAMs) and enhanced viral virulence (22). Conversely, mutations at positions T586A and S592T were found to reduce viral replication efficiency and attenuate pathogenicity in piglets (36). Our investigation of PRRSV non-structural proteins reveals a significant correlation between viral replicases and pathogenicity.

In summary, utilizing the PRRSV mini-replicon platform, this study reveals a molecular mechanism whereby adaptive mutations in conserved residues of nsp9 and nsp10 drive PRRSV evolution through modulation of RNA synthesis efficiency. These substitutions likely contribute to strain-specific replication phenotypes by fine-tuning the catalytic activity and conformational dynamics of RdRp and helicase complexes. However, further investigation is required to elucidate the impact of these crucial mutations on viral replication kinetics and pathogenicity both *in vitro* and *in vivo*.

## Experimental procedures

### Cells and viruses

BHK-21 and MARC-145 cells were purchased from ATCC and routinely cultured in DMEM supplemented with 10% FBS and 1% penicillin/streptomycin at 37 °C with 5% CO_2_. PRRSV recombinant strain rHP-PRRSV/SD16/TRS6-EGFP was provided by Professor Enmin Zhou from Northwest A&F University. pCDNA3.1(+), pCMV-HA, pCI-neo, pGL3-Basic, and pRL-TK were stored in our laboratory. Mouse polyclonal antibodies against PRRSV-N protein were generated in our laboratory.

### Antibodies and plasmids

The primary antibodies used in this study were: rabbit anti-PRRSV-N polyclonal antibody (generated in our laboratory), rabbit anti-Fluc luciferase polyclonal antibody (BIOSS, bsm-33318M), mouse anti-β-Actin monoclonal antibody (Tianjin Sungene, DKM9001L), and mouse anti-GAPDH monoclonal antibody (Tianjin Sungene, DKM9002). The secondary antibodies were HRP-labeled goat anti-mouse IgG (Tianjin Sungene, LK2003L) and Alexa Fluor 488-labeled goat anti-mouse IgG (Beyotime, A0428). Eukaryotic expression vectors pCDNA3.1(+), pCMV-HA, pCI-neo, pGL3-Basic, and pRL-TK were stored in our laboratory. The genes encoding PRRSV non-structural proteins (nsp8-9, nsp10, nsp11, and nsp12) were amplified from GD and XM-2020 strains and cloned into the pCI-neo vector. A series of nsp8-9, nsp10, and nsp12 mutants were constructed using site-directed mutagenesis. Primers are listed in the supporting information text.

## Construction and design of PRRSV replicon systems

### Generation of full-length PRRSV replicons

To investigate the genomic replication and transcription mechanisms of PRRSV, we established a reporter gene-carrying replicon plasmid (Rep-PRRSV) based on a reverse genetics platform using the GD strain as the backbone. This replicon system retains the critical elements necessary for viral subgenomic mRNA synthesis, including the 5′ and 3′ untranslated regions (UTRs), ORF1a/b replicase genes, ORF7, and a dual reporter cassette (Fluc-T2A-RFP) under the control of TRS6 to quantitatively assess subgenomic mRNA transcription. Additionally, we positioned a CMV promoter at the 5′ terminus of the replicon construct, while the 3′ terminus was systematically appended with the Hepatitis delta virus ribozyme (HdvRz) sequence followed by the BGH polyA termination signal. Furthermore, the post-translationally processed products of ORF1b translation—nsp9, nsp10, nsp11, and nsp12—constitute core components of the PRRSV replicase and play critical roles in viral genomic replication and transcription, while also serving as key hub proteins connecting the PRRSV nsp interaction network. Therefore, we constructed a replication-deficient control replicon (Rep-ΔORF1b) with ORF1b deletion based on Rep-PRRSV, as shown in Figure 1*A*. To evaluate these replicon systems, we primarily assessed the autonomous RNA synthesis activity by detecting N protein expression and Fluc/RFP fluorescence.

### Development of minimalist PRRSV replicon system

Furthermore, despite our successful establishment of comprehensive PRRSV replicons encompassing the complete nsp gene repertoire (ORF1a and ORF1b), the considerable genomic length inherently constrained their utility for mechanistic investigations, particularly those focused on replicase mutations. To enhance experimental tractability, we engineered a PRRSV mini-replicon system, conceptually aligned with SARS-CoV-2 mini-replicon architectural principles (29, 30), designed to recapitulate viral RNA biosynthetic pathways with minimal but sufficient genetic elements. Utilizing the Rep-PRRSV plasmid backbone, we strategically retained the CMV promoter, 5′UTR, Fluc reporter gene, N gene, and 3′UTR to generate the subgenomic replicon designated sg-Rep-PRRSV, as depicted schematically in Figure 3*A*. This minimalist replicon effectively mimics viral genomic replication and transcriptional processes when facilitated by the mini-RTC complex.

### Assembly of the minimal replication-transcription complex (mini-RTC)

Based on the conserved assembly pattern of the replication-transcription complex (RTC) among PRRSV and other nidoviruses, this study selected nsp9 (RdRp) along with its closely associated proteins nsp10, nsp11, and nsp12 to construct a quaternary “mini-RTC” system. As nsp8 has been demonstrated to function as an N-terminal extension of nsp9 (31), we eliminated the ribosomal frameshift site between these two proteins, generating a nsp8-9 fusion protein. The genes encoding nsp8-9 (with the ribosomal frameshift site removed), nsp10, nsp11, and nsp12 were amplified from GD and XM-2020 viral strains and subsequently cloned into pCI-neo eukaryotic expression vectors, yielding four independent nsp expression plasmids. These plasmids were co-transfected into BHK-21 cells at optimized ratios (nsp8-9:nsp10:nsp11:nsp12 = 2:2:1:1) to recapitulate the assembly of a viral mini-replication-transcription complex (mini-RTC) in a cellular context.

### Construction of key non-structural protein mutants

Site-directed mutagenesis was employed to generate a series of nsp8-9, nsp10, and nsp12 mutant plasmids. Primers were designed with target mutation sites centrally positioned and flanked by 18 to 25 bp homologous arms at both termini. Gibson Assembly was utilized to combine the amplified mutated fragments with linearized vectors at a 2:1 M ratio, with the reaction mixture incubated at 50 °C for 40 min. For transformation, 10 μl of the ligation product was introduced into Takara HST08 competent cells and subsequently cultured overnight at 37 °C on LB agar plates containing appropriate selective antibiotics. Positive clones were isolated the following day for plasmid extraction and sequence verification. All primer sequences utilized in this study are summarized in the supporting information text.

### Cell transfection

Transfection was performed following the manufacturer's protocol for Turbofect transfection reagent. MARC-145 cells cultured in 12-well plates were transfected upon reaching approximately 75% confluence. The culture medium was replaced with serum-free Opti-MEM 2 h prior to transfection. For each well, 1.5 μg of plasmid DNA was diluted in 200 μl of serum-free Opti-MEM and gently homogenized. Subsequently, 4 μl of Turbofect reagent was added to the diluted DNA solution, immediately vortexed, and incubated at room temperature for 15 min. The resulting transfection complexes were added dropwise to the cell monolayer, and the plate was gently agitated to ensure uniform distribution. Transfected cells were maintained in culture for 24 to 36 h before subsequent analyses were performed.

### Western blot analysis

Protein samples were lysed in radioimmunoprecipitation assay (RIPA) buffer supplemented with 1% phenylmethylsulfonyl fluoride (PMSF) and a phosphatase inhibitor cocktail. The lysates were subsequently boiled for 10 min in 5×sodium dodecyl sulfate-polyacrylamide gel electrophoresis (SDS-PAGE) loading buffer. The proteins were separated by 10% SDS-PAGE and transferred onto nitrocellulose membranes. The membranes were blocked with 5% skim milk for 1 h and incubated with primary antibodies at 4 °C overnight, followed by horseradish peroxidase (HRP)-conjugated secondary antibodies for 1 h at room temperature. The protein bands were detected using an enhanced chemiluminescence (ECL) detection system.

### Dual-luciferase reporter assay

Luciferase activity was measured using the Dual-Luciferase Reporter Assay System (Promega) with a GloMax luminometer. Replicon plasmids were transfected into MARC-145 or BHK-21 cells seeded in 12-well plates using Lipofectamine 3000, with three biological replicates per group. At 12, 24, 36, and 48 h post-transfection, cells were lysed with 200 μl of 1×Passive Lysis Buffer (Promega). Cell lysates (20 μl) were transferred to a 96-well white plate, and firefly and Renilla luciferase activities were measured using LAR II and Stop&Glo reagents (Promega), respectively. The relative luciferase activity was calculated as the ratio of firefly to Renilla luciferase activity and normalized to that of the negative control (set as 1.0).

### Transmission electron microscopy analysis

MARC-145 cells at 80% confluence were co-transfected with replicon plasmids using Lipofectamine 3000. At 24 h post-transfection, cells were scraped and collected by centrifugation at 1000*g* for 5 min. Samples were fixed with 2.5% glutaraldehyde and 1% osmium tetroxide, dehydrated with ethanol and acetone, and infiltrated with Epon 812 epoxy resin. Samples were polymerized, sectioned (55–65 nm), stained with uranyl acetate and lead citrate, and observed under a transmission electron microscope.

### Quantitative real-time PCR

Total RNA was isolated from cells using TRIzol reagent (Takara). Reverse transcription was performed using the HiScript III RT SuperMix reverse transcription kit (Vazyme Biotech). Quantitative reverse transcription polymerase chain reaction (RT-qPCR) was performed using an ABI 7500 Real-Time PCR system (Applied Biosystems) with TB Green Premix Ex Taq II (Takara). The relative expression levels were normalized to chicken β-actin and calculated using the 2^−ΔΔCT^ method. Primers are listed in Table 1. All RT-qPCR experiments were performed with three technical replicates.

**Table 1:** Primers used in this study

Target gene	Primer sequence (5′-3′)
PRRSV-N-F	CTAGCGACTGAAGATGAC
PRRSV-N-R	GCAAACTAAACTCCACAG
sg mRNA7.1-F	GGAGTTTAGGGATTTGTC
sg mRNA7.1-R	ATATTTAACAAGGTTCACC
sg mRNA7.2-F	CACTGCTTTACGGTCTCTC
sg mRNA7.2-R	TCTGTTGGGCGATAATCTT
sg mRNA-Fluc-F	ACTGCTTTACGGTCTCTCC
sg mRNA-Fluc-R	CTCTCCAGCGGTTCCATCT
ORF1ab-F	ACAGGGATTCAGTGTGGGCA
ORF1ab-R	ATTCTTTTCAGTTTCTCCAG
β-actin-F	ACCACCATGTACCCAGGCAT
β-actin-R	GGACTCGTCGTACTCCTGCT

### Flow cytometry detection of reporter gene expression

BHK-21 cells at 75% confluence were trypsinized, collected, and adjusted to 10^6^-10^7^ cells/ml. Cells were fixed with 4% paraformaldehyde, permeabilized with 0.1% Triton X-100, and blocked with 5% BSA. Mouse anti-Fluc antibody (1:100) was added and incubated at 4 °C for 90 min. After washing, Alexa Fluor 488-labeled goat anti-mouse IgG (1:200) was added and incubated at 4 °C for 60 min. Cells were resuspended in PBS and analyzed by flow cytometry. The proportion of Fluc-positive cells and mean fluorescence intensity (MFI) were calculated using FlowJo software.

### Laser confocal microscopy observation

BHK-21 cells at 75% confluence were trypsinized with 0.25% trypsin-EDTA for 5 min, collected, and adjusted to 1 × 10^6^-1 × 10^7^ cells/ml. Cells were fixed with 4% paraformaldehyde for 15 min at room temperature, permeabilized with 0.1% Triton X-100 for 10 min, and blocked with 5% BSA in PBS for 30 min. Mouse anti-Fluc antibody (1:100 diluted in PBS containing 1% BSA) was added and incubated at 4 °C for 90 min. After washing three times with PBS, Alexa Fluor 488-labeled goat anti-mouse IgG (1:200 diluted in PBS containing 1% BSA) was added and incubated at 4 °C for 60 min. Cells were washed three times with PBS, resuspended in 500 μl PBS, and analyzed using a flow cytometer. The proportion of Fluc-positive cells and MFI was analyzed using FlowJo software.

## Statistical analysis

All statistical analyses and graphs were generated using GraphPad Prism version 10.0. Data are presented as mean ± standard deviation (SD) from at least three independent experiments. Statistical comparisons between two groups were performed using an unpaired two-tailed Student's *t* test. Differences were considered statistically significant at ∗*p* < 0.05, ∗∗*p* < 0.01, ∗∗∗*p* < 0.001, and ∗∗∗∗*p* < 0.0001.

## Data availability

All data described are contained within the article.

## Supporting information

This article contains supporting information: Supporting Information Text. xlsx.

## Conflict of interest

The authors declare that they have no conflicts of interest with the contents of this article.
